# A Fragile Zero Watermarking Scheme to Detect and Characterize Malicious Modifications in Database Relations

**DOI:** 10.1155/2013/796726

**Published:** 2013-06-02

**Authors:** Aihab Khan, Syed Afaq Husain

**Affiliations:** ^1^Department of Computing & Technology, Iqra University, Islamabad 44000, Pakistan; ^2^Faculty of Computer Science & IT, King Faisal University, Ahsaa 31982, Saudi Arabia

## Abstract

We put forward a fragile zero watermarking scheme to detect and characterize malicious modifications made to a database relation. Most of the existing watermarking schemes for relational databases introduce intentional errors or permanent distortions as marks into the database original content. These distortions inevitably degrade the data quality and data usability as the integrity of a relational database is violated. Moreover, these fragile schemes can detect malicious data modifications but do not characterize the tempering attack, that is, the nature of tempering. The proposed fragile scheme is based on zero watermarking approach to detect malicious modifications made to a database relation. In zero watermarking, the watermark is generated (constructed) from the contents of the original data rather than introduction of permanent distortions as marks into the data. As a result, the proposed scheme is distortion-free; thus, it also resolves the inherent conflict between security and imperceptibility. The proposed scheme also characterizes the malicious data modifications to quantify the nature of tempering attacks. Experimental results show that even minor malicious modifications made to a database relation can be detected and characterized successfully.

## 1. Introduction

Digital watermarking is a class of information hiding technique that provides measures for copyright protection, broadcast monitoring, covert communication, copy control, tamper, and integrity proof of digital assets. The watermarking techniques were primarily proposed for multimedia content [1–4]; however, in the last decade, the research community has extended these techniques to relational databases for its copyright protection, temper detection, and integrity proof. Most of the existing watermarking schemes for relational databases [5–20] introduce intentional errors or distortions as marks in the underlying data with some error tolerance so that it does not have a significant impact on the usefulness of data. However, this results in degrading data quality as the integrity of a relational database is violated. A large collection of real-world datasets has a strong usability constraint that disallows any permanent distortions or intentional errors. For example, the safety critical datasets are designed to minimize errors rather than to introduce intentional errors. Similarly, a business application may require that local properties like item-cost, ordered-quantity, and so forth, are preserved as well as global properties like natural join between item and sales, employees and department, and so forth. Moreover, in business datasets, the semantic constraints are not violated, like dissimilarity in attribute value for two similar transactions [21]. Query processing is sensitive due to selection criteria and has well-defined semantics; therefore, the watermarking schemes that introduce distortion into the database original content are not appropriate for certain applications.

Based on the intent of marking, the watermarking schemes presented in the literature can be categorized into robust and fragile schemes. The robust schemes [5–16] are aimed at copyright protection, whereas the fragile schemes [17–25] are used for tamper detection and integrity proof of database relations. Most of the robust schemes for copyright protection [5–16] introduce distortions into the database original content which affects data integrity and usability. These robust schemes may work for numeric [5–10] and categorical attributes [11, 12] of relational databases to embed watermarks. Some techniques embed meaningless bit pattern [5, 6]; whereas in other techniques meaningful bit patterns like image [13–15] and owner's speech [16] are used as watermarks for embedding in relational databases. In data sales environment, some of these robust schemes are extended to fingerprinting domain for unique identification of each buyer and also for traitor detection [21, 26–28]. Compared with the robust schemes, the fragile watermarking schemes are not adequately addressed and relatively little work is available for integrity proof of relational databases [20]. In this paper, we focus on fragile watermarking schemes for temper detection and integrity proof of database relations. 

The initial work on fragile watermarking schemes can be found on images [29–31], which is extended to audio [32, 33] and video [3, 34] schemes. Recently, the importance of other data domains is recognized and fragile schemes for text [35, 36] and relational databases [17–20, 22–25] are proposed. Like robust schemes, most of the fragile schemes for relational databases [17–20] introduce distortion into the database original contents that degrades data quality and also affects data usability. These schemes are based on the content characteristics of database relation itself to create a secure hash (used as a watermark) which is stored in Least Significant Bits (LSBs) of database original contents, thus introducing distortion. 

A fragile watermarking scheme presented by Guo et al. [17] detects malicious modifications made to a database relation. In their scheme, the watermark generation is based on the content characteristics of the database relation itself. The generated watermarks are embedded in at most two LSBs of all attributes in the database relation that introduces considerable distortion in the database original contents. The fragile scheme presented by Khataeimaragheh and Rashidi [18] is also a distortion-based scheme for integrity proof of database relations. Like [17], the watermarks are embedded in at most two LSBs of all attributes in the relation that forms a two-bit watermark grid. The fragile scheme presented by Iqbal et al. [19] logically partitions the database relation into three groups and generates self-constructing fragile watermark information from each group. The generated watermarks are embedded at LSBs of numerical attributes in each group of a database relation which introduces distortion in database original contents. Prasannakumari [20] presented a fragile scheme for temper detection in database relations. This technique also introduces distortion as it inserts a fake attribute in database relation to act as a watermark. The data values for the newly inserted attribute are determined by applying aggregate function on original database content.

Beside distortion-based techniques, some researches also presented distortion free fragile watermarking schemes [22–25] for integrity proof of database relations. The main feature of these schemes is that the watermark embedding in actual fact is the tuples or attributes reordering based on the content characteristics of database relation. A fragile scheme proposed by Li et al. [22] detects and localizes malicious modifications made to the database relations. Their scheme partitions the database relation into disjoint groups and the watermark is embedded and verified in each group independently. In their scheme, the watermark is embedded as tuple reordering and the order of each tuple pair in group is changed or unchanged depending on the tuple hash values and the corresponding group hash value. Though their technique does not introduce any distortion in the database relation, but it works only for categorical data type. Kamel [23] presented a fragile scheme to protect the integrity of database relations. Their scheme divides the database relations in groups and each group is marked independently. As in [22], the watermark embedding is reordering of tuples in each group that corresponds to the value of some secret watermark. The fragile scheme proposed by Bhattacharya and Cortesi [24] detects malicious modifications in database relations having categorical attributes. Their scheme divides the database relation into groups on the basis of categorical attribute values. Like [22, 23], tuple hash value is used to obtain a watermark as permutation of tuples. A fragile zero watermarking scheme is presented by Hamadou et al. [25] for authentication of database relations. Their technique is distortion-free and is based on attribute reordering method. Initially, the attributes of database relation are virtually sorted on hash values of attribute names to define a secret initial order of attributes. For each attribute in database relation, the Most Significant Bits (MSBs) are extracted and used for watermark generation. The generated watermark is then registered with the Certification Authority (CA) for certification purpose. As their technique is based on virtual sorting of attributes by their names, so any change in attribute name by attacker would fail the temper detection process. 

In the previous discussion, we have identified two important issues in existing fragile watermarking schemes. First, the fragile schemes are distortion based [17–20] that inevitably degrade data integrity and thus affect data usability; therefore, these schemes are not applicable to non-error-tolerant data like safety critical datasets, and so forth. Second, though there exist some fragile schemes like [22–25] that are distortion-free, but the watermarking approach is based on reordering of tuples or attributes; so, they are vulnerable to sorting attacks. Also, if the modification is small, such that, it does not affect the order of tuples, the temper detection would fail. To address these issues, we propose a fragile scheme based on zero watermarking approach that does not modify any part or properties of the database relations itself; therefore, the proposed scheme assures imperceptibility and overcomes weaknesses like data integrity and data usability in existing fragile watermarking schemes. Also, the proposed scheme is independent of tuple ordering as well as attributes ordering and naming, so it is not vulnerable to sorting attacks. The watermark generation in the proposed scheme is based on algorithmically evaluating the local characteristics of database relation like frequency distribution of digit count, length and range of data values. This enables us to characterize the malicious data modifications on parameters like the fraction of digit, length and range of data values attacked, the type of attack (insertion, deletion, or update), and the effect of attack (low to high, high to low, or no change) on data values. Also, to the best of our knowledge, there is no such distortion-free fragile watermarking scheme that can characterize the tempering attacks, that is, the nature of tempering. Experimental results show that the proposed scheme can detect and characterize malicious data modifications successfully.

## 2. Materials and Methods

In this section, we present our proposed fragile zero watermarking scheme to detect and characterize malicious modifications made to a database relation. The proposed scheme exhibits the following important properties of a fragile watermarking system as discussed in [17].

1. *Fragility. *The proposed scheme is designed to be fragile; that is, if there are any malicious data modifications, the embedded watermark is not detectable (destroyed).
2. *Imperceptibility.* As the proposed scheme is based on zero watermarking approach, it does not introduce any distortion in the underlying data; therefore, the embedded watermark is invisible or imperceptible.
3. *Key-Based System. *The watermark generation and verification in the proposed scheme is a key-based system. Also, to detect and characterize malicious data modifications, a secret key is required.
4. *Blindness*. In the proposed scheme, the original database relation is not required to detect and characterize malicious data modifications.
5. *Tuple and Attribute Ordering. *The existing fragile schemes are based on tuple ordering [22–24] and attribute ordering and naming [25]. The proposed scheme is independent of tuple and attributes ordering so it is not vulnerable to sorting attacks.
6. *Characterization. *The proposed scheme not only detects but also characterizes the malicious data modifications in database relation to quantify the nature of tempering attacks.

### 2.1. Watermark Generation

**Figure 1:**
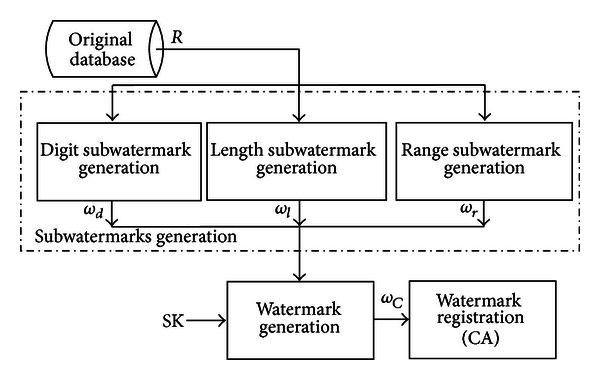
Proposed model for watermark generation and registration.

Let *R* be a database relation with primary key PK and *ν* attributes denoted by *R*(PK, *A*1, *A*2,…, *A*_*ν*_). The watermark generation in the proposed scheme is based on the content characteristics of numeric data values, so we assume that some attributes of the database relation are numeric.  Figure 1 shows the watermark generation process that comprises of subwatermark generation for digit count, length, and range of data values. The generated watermark is registered with the Certification Authority (CA) for certification purpose.  Table 1 presents the list of notations used in our algorithms and discussion. 

The algorithm for watermark generation is presented in Algorithm 1. At lines 1–3, the digit, length, and range of data values in a database relation are algorithmically evaluated to generate the subwatermarks as presented in Algorithms 2–4. These subwatermarks are then used to generate a database relation watermark *ω*_*R*_ as shown at line 4. At line 5, the relation watermark *ω*_*R*_ is encrypted with a secret key SK known only to the database owner. We assume that the secret key is selected from large key space such that it is computationally infeasible for attacker to guess a key. At lines 6-7, the encrypted relation watermark *Eω*_*R*_ is concatenated with owner Id along with date and time stamp to generate a watermark certificate *ω*_*C*_, which is then registered with the CA before publishing the database for certification purpose. 

Algorithm 2 generates a digit subwatermark which is based on digit frequency for all data values present in adatabase relation. At lines 1–3, the length of each data value is determined which is then used to extract the individual digits as shown at lines 4-5. Lines 6-7 compute the frequency of each digit and the total number of digits present in the database relation. At line 11, the relative frequency of each digit *rfd*_*i*_ is determined which is then used to generate a digit subwatermark *ω*_*d*_ as shown at line 13. At lines 15-16, the digit subwatermark *ω*_*d*_ is concatenated with total digit count and is returned to the watermark generation algorithm. It is to be noted that the digit subwatermark is composed of each digit relative frequency *rfd*_*i*_ and the total count of all digits. In fact, this information is used for characterization of attacks as discussed in Section 3. 

The subwatermark generation for length of data values in a database relation is presented in Algorithm 3. At lines 1–3, the length of each data value is determined. Lines 4-5 determine the frequency for each length of data values and the total count of data values length present in the database relation. At line 9, the relative frequency for each length of data value *rfl*_*j*_ is computed which is then used to generate length subwatermark *ω*_*l*_ as shown at line 10. At lines 12-13, the length subwatermark *ω*_*l*_ is concatenated with total length count and is returned.

Algorithm 4 presents the algorithm for subwatermark generation for range of data values in a database relation. At line 1, different data ranges are defined in which the data value of a database relation may fall. It is to be noted that the defined data ranges may be adjusted as per the nature of data values in the database relation and also for more precise characterization of malicious data modifications, as discussed in Section 3. Lines 1–3 determine the attribute value, within each tuple. Lines 5–13 determine the frequency for different data ranges in which the data value may fall and the total number of data ranges present in the database relation. At lines 16-17, the relative frequency for each range of data value *rfr*_*k*_ is computed, which is then used to generate range subwatermark *ω*_*r*_. Lines 19-20 show that the range subwatermark *ω*_*r*_ is concatenated with total range count and is returned.

### 2.2. Watermark Verification

**Figure 2:**
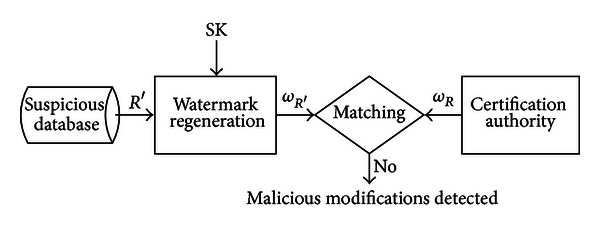
Proposed model for detection of malicious tempering.

Figure 2 shows the model for detection of malicious modifications in suspicious database relation *R*′. For detection of malicious data modifications, the relation watermark *ω*_*R*_′ is regenerated for suspicious database relation *R*′ and compared with the relation watermark *ω*_*R*_ registered at CA; if both watermarks are different then the suspicious database relation *R*′ is considered as a tempered relation. 

The algorithm for watermark detection is presented in Algorithm 5. At line 1, the watermark *ω*_*R*_′ is generated by using Algorithm 1 for suspicious database relation *R*′. The watermark certificate *ω*_*C*_ which is already registered at CA is used to extract database relation watermark *ω*_*R*_ as shown at lines 2–4. At lines 5–10, each digit of *ω*_*R*_ is compared with the corresponding digit of *ω*_*R*_′ and match_count is incremented on each successful match. At line 9, the total_count is computed to know the number of digits tested. At lines 11-12, the WAR (Watermark Accuracy Rate) and WDR (Watermark Distortion Rate) are computed. If the distortion exists in the suspicious database relation *R*′, then *R*′ is rejected as a tempered relation with distortion rate WDR as shown at lines 13–15.

The algorithm for characterization of malicious data modifications is presented in Algorithm 6. At line 2, the relative frequency of each digit *rfd*_*i*_ is extracted from digit subwatermark *ω*_*d*_ as *ω*_*d*_⊆*ω*_*R*_ and *ω*_*R*_ is already registered at CA. The frequency distribution of each digit *fd*_*i*_ in relation *R* is determined at line 3. At line 4, the frequency distribution of each digit *fd*_*i*_′ for suspicious database relation *R*′ is determined. The change in frequency distribution of each digit Δ*fd*_*i*_ is computed at line 5 and the fractional change in each digit Δ*ℱd*_*i*_ is determined at line 6. The computed value of Δ*ℱd*_*i*_ is then used to characterize the malicious modifications made to the database relation *R*. For example, if Δ*ℱd*_*i*_ is zero, then the suspicious relation *R*′ is not tempered. A positive Δ*ℱd*_*i*_ indicates that *ℱ* fraction of digit *d*_*i*_ is maliciously inserted by attacker as an attempt to transform low data values to high in database relation *R*. Similarly, a negative Δ*ℱd*_*i*_ indicates that *ℱ* fraction of digit *d*_*i*_ is maliciously deleted by attacker as an attempt to transform high data values to low in database relation *R*. At lines 8–14 and 15–21, a similar method as discussed earlier is used to determine Δ*ℱl*_*j*_ and Δ*ℱr*_*k*_ to characterize the attacks on length and range of data values in database relation *R*. The characterization of malicious data modifications is further elaborated in Section 3.2 with experimental results.

## 3. Results and Discussion

Suppose that Alice is the database owner and she has used the proposed algorithms along with the secret key to generate a watermark for the database relation *R*. The attacker Mallory for his own nefarious objectives may attempt to make malicious modifications in Alice watermarked database relation. We conducted our experiments in Microsoft Visual Basic and Microsoft Access, on 3.2 GHz Intel core i3 CPU with 2 GB of RAM. The proposed watermarking scheme is evaluated on a real-life dataset namely Forest Cover Type data set, available at UCI Machine Learning Repository [37]. This dataset has 581,102 tuples, each with 10 integer attributes, 44 Boolean attributes, and 1 categorical attribute. In our experiments, we have used all 10 integer attributes. It is to be noted that in robust watermarking schemes, the aim of Mallory is to destroy the Alice watermark without affecting the database relation, whereas in fragile schemes, Mallory attempts to make malicious modifications in Alice watermarked database relation without affecting the watermark. The experimental results presented in this section show that the watermark is adversely affected by even minor malicious data modifications; therefore, the generated watermark is fragile. 

### 3.1. Detection of Malicious Modifications

In this set of experiments, we randomly introduce malicious modifications in Forest Cover Type data set [37]. As discussed in Algorithm 5, these malicious modifications are detected by generating the watermark for the suspicious database relation *R*′ to obtain *ω*_*R*_′, which is then compared with the registered watermark *ω*_*R*_ to determine the WAR (Watermark Accuracy Rate) and WDR (Watermark Distortion Rate). 

Table 2 shows the WAR and WDR for the malicious insertions made to the database relation with different attack rates. For example, when 10% of the fake but similar tuples are randomly inserted into the database relation *R*, the WDR is found to be high and malicious insertions are detected with low WAR.

Tables 3-4 show similar results as of insertion attack for malicious deletions and updates made to the database relation *R*. 

**Figure 3:**
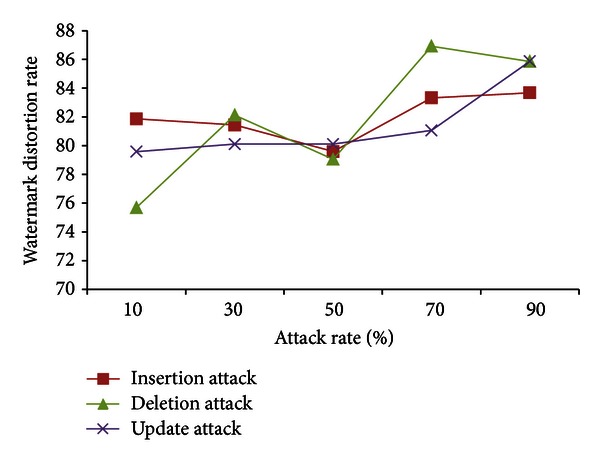
Watermark distortion rate for malicious insertion, deletion, and update of tuples with different attack rates (*n* = 10^6^).

Figure 3 summarizes the insertion, deletion, and update attacks and shows that the WDR is always high for different volume of malicious data modifications. 

In another set of attacks, we simultaneously perform malicious insertion, deletion, and update of tuples with different attack rates in database relation *R*.  Table 5 shows the WDR for this set of attack.

The experimental results presented in Tables 2–5 show that the malicious modifications are always detected and fragility of the registered watermark *ω*_*R*_ is observed for even low volumes of attack. The WAR is low and WDR is high for different volume of malicious insertions, deletions, and updates made to the database relation. The low WAR indicates the extent to which the database relation has been attacked, whereas the high WDR indicates that the database relation has been tampered and is not authentic. The accuracy of watermark is adversely affected even with minor malicious data modifications and the watermark fragility proves that the database relation has been attacked.

### 3.2. Characterization of Malicious Modifications

One of the important features of the proposed watermarking scheme is to characterize the malicious modifications made to the database relations. As discussed in Algorithm 1, the watermark generation is based on the content characteristics of database relation itself which enable us to characterize the malicious data modifications. Algorithm 6 elaborates the algorithm for characterization of malicious data modifications by evaluating the fractional change in each digit Δ*ℱd*_*i*_, length Δ*ℱl*_*j*_ and range Δ*ℱr*_*k*_ of data values in the tempered database relation *R*′.

We have conducted experiments for both random and deterministic attacks for characterization of malicious data modifications. In random tempering attacks, we randomly attack the digit frequency, length, and range of data values in the database relation, whereas in deterministic attacks, the attack is performed with the specific attack rates. The random tempering attacks are presented in this section and the results of detailed deterministic attacks are shown in the Appendix for reference.

#### 3.2.1. Attacks on Digit Frequency

In this set of attacks, Mallory randomly performs malicious insertion, deletion, and update attacks on digit frequency in Alice's watermarked relation *R*. For example, in insertion attack, Mallory may attempt to maliciously insert some digits in *R*.  Table 6 shows the experimental results obtained for characterization of malicious insertion attack on digits 9 and 0 as discussed in Algorithm 6. A positive value of Δ*ℱd*_*i*_ indicates that *ℱ* fraction of digits 9 and 0 is maliciously inserted by Mallory in the database relation *R*. The characteristic of this attack is an attempt to relatively increase the low data values to high in database relation *R* as an increase of 35.84% and 24.42% is observed in Δ*ℱd*_*i*_ of digits 9 and 0, respectively. As the other digits are not attacked, so Δ*ℱd*_*i*_ is zero for digits 1–8 and there is no change in the digit frequency Δ*fd*_*i*_ of these digits. This characteristic of attack, when combined with the nature of data, may provide useful information about the attacker intention. For example, in the product sales environment, these malicious insertions indicate that the attacker may have attempted to increase the low volume and amount of product sales. 

Table 7 shows the result for random malicious deletions of digits 9 and 0 made to the database relation *R*. A negative value of Δ*ℱd*_*i*_ indicates that *ℱ* fraction of digits 9 and 0 is maliciously deleted by the attacker. The characteristic of this attack is an attempt to relatively decrease the high data values to low in the database relation *R*. In this attack, 14.70% of digit 9 and 12.44% of digit 0 are randomly deleted from the database relation. As the other digits are not deleted, so Δ*ℱd*_*i*_ is zero for digits 1–8.  Table 8 shows similar result for random malicious update for digits 9 and 0 made to the database relation. In this attack, digits 9 and 0 are randomly replaced with some other digits, so the digit frequency Δ*fd*_*i*_ of digits 9 and 0 is decreased (high to low), where as the digit frequency Δ*fd*_*i*_ of digits 1–8 is increased (low to high). 

**Figure 4:**
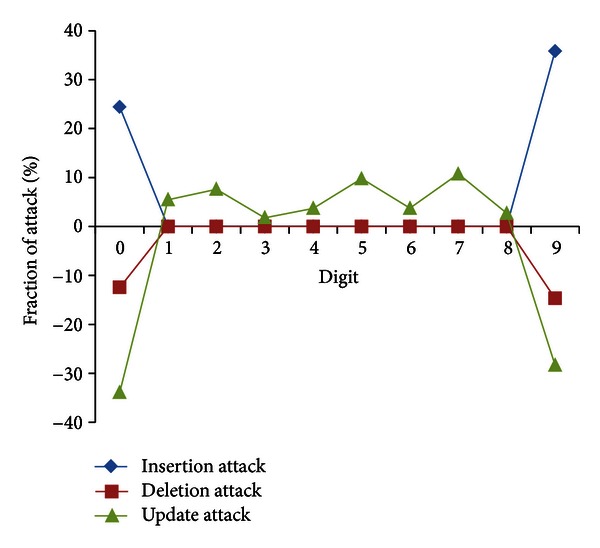
Characterization of malicious insertion, deletion, and update attacks on digits 9 and 0 of data values.

Figure 4 summarizes the malicious insertion, deletion, and update attacks on digits 9 and 0. The insertion attack shows a positive increase (low to high) on attacked digits, where as a negative trend (high to low) is observed in attacked digits for deletion attack. In update attack, both negative (high to low) and positive trends (low to high) are observed for attacked and unattacked digits, respectively.

In another set of attacks, we randomly insert, delete and update 10% (lower bound) and 90% (upper bound) of the tuples from the database relation *R*.  Table 9 shows the effect on fractional change in digit frequency Δ*ℱd*_*i*_ for each digit. It is to be noted that, in insertion attack, a *k* fraction of positive trend (low to high) is being observed in each digit frequency of database relation *R*. For example, when 10% of similar tuples are inserted in database relation, an increase of approximately 10% is being observed in Δ*ℱd*_*i*_ for each digit of database relation. Similarly, in deletion attack, a *k* fraction of negative trend (high to low) is observed in Δ*ℱd*_*i*_ for each digit of database relation. In update attack, no specific trend is observed in Δ*ℱd*_*i*_ as *k* fractions of digits are randomly replaced by some other digits.

It is to be noted that the attack on digit frequency (as discussed above) can be characterized on parameters like the digits being attacked, the fraction of each digit attacked, the type of attack (insertion, deletion, or update) on each digit, and the effect of attack (low to high, high to low, or no change) on data values. 

#### 3.2.2. Attack on Length of Data Values

In this set of attacks, Mallory randomly performs malicious insertion, deletion, and update attacks on length of data values.  Table 10 shows the experimental result for characterization of malicious insertion on data values of length 3 in the database relation *R*. A positive value of Δ*ℱl*_*j*_ indicates that *ℱ* fraction of length *l*_*j*_ is maliciously inserted in the database relation *R*. The characteristic of this attack is to relatively increase the low data values to high as an increase of 18.27% is observed in Δ*ℱl*_*j*_ for data values of length 3. Also, Δ*ℱl*_*j*_ is zero for lengths 1, 2, and 4, which shows that the data values of these lengths are not attacked. 

Table 11 shows result of random malicious deletion for data values of length 3. As in deletion of digit frequency attack, a negative value of Δ*ℱl*_*j*_ indicates that *ℱ* fraction of length *l*_*j*_ is maliciously deleted with characteristic of decreasing high data values to low in database relation. Also, as in malicious insertion, the Δ*ℱl*_*j*_ is zero for lengths 1, 2, and 4, which indicates that the data values of these lengths are not deleted.  Table 12 shows results for malicious updates on data values of length 3. In this attack, the data values of length 3 are randomly replaced by lengths 1, 2, and 4. This attack shows a decrease in Δ*ℱl*_*j*_ for length 3, where as the Δ*ℱl*_*j*_ for lengths 1, 2, and 4 is increased. 

**Figure 5:**
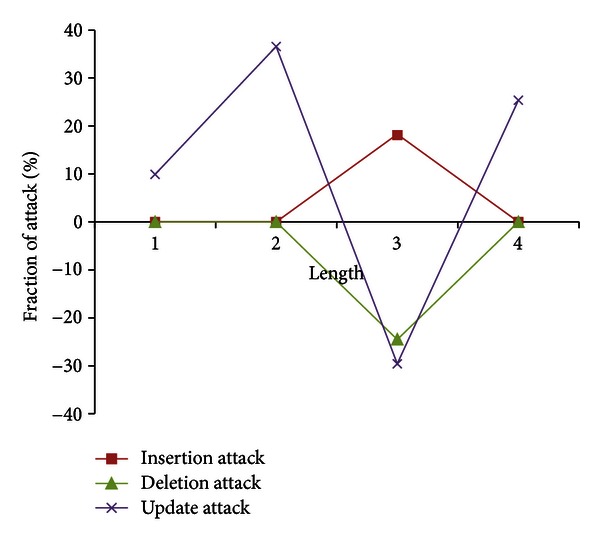
Characterization of malicious insertion, deletion, and update attacks on length 3 of data values.

Figure 5 summarizes the malicious insertion, deletion, and update attacks on length 3 of data values. The insertion attack shows a positive increase (low to high) in attacked length, where as a negative trend (high to low) on attacked length is observed in deletion attack. In modification attack, a negative trend (high to low) is observed on attacked length, where as a positive trend (low to high) is observed on un-attacked length of data values. 

Table 13 shows the effect on fractional change in length frequency Δ*ℱl*_*j*_, when 10% (lower bound) and 90% (upper bound) of tuples are maliciously inserted, deleted, and updated in the database relation. In insertion attack, the fractional change in length frequency Δ*ℱl*_*j*_ has a *k* fraction of positive trend (low to high) for each length of data values. Similarly, in deletion attack, a *k* fraction of negative trend (high to low) is observed for each length of data values. For example, when 10% of tuples are randomly deleted from a database relation, a decrease of approximately 10% is observed in Δ*ℱl*_*j*_ for each length of data values. The update attack does not show any specific trend as *k* fraction of different length of data values are randomly replaced by some other length of data values.

It is to be noted that the attack on length of data values can be characterized on parameters like the length of data values being attacked, the fraction of each length of data values attacked, the type of attack (insertion, deletion, or update), and the effect of attack (low to high, high to low, or no change) on each length of data values. 

#### 3.2.3. Attack on Range of Data Values

In this set of attacks, Mallory randomly performs insertion, deletion, and update attack on range 1, that is, (100–999) of data values present in the database relation *R*.  Table 14 shows the experimental results for characterization of malicious insertion for range 1 of data values. The characteristic of this attack is to relatively increase the low data values to high as an increase of 17.33% is observed in Δ*ℱr*_*k*_ for range 1 of data values. The Δ*ℱr*_*k*_ for range 0 and 2 is zero as the data values of these ranges are not attacked.

Table 15 shows the results of random malicious deletion for data values of range 1. As in deletion of digit frequency attack, a negative value of Δ*ℱr*_*k*_ indicates that *ℱ* fraction of range 1 is maliciously deleted with characteristic of transforming high data values to low in database relation *R*. As the data values of ranges 0 and 2 are not attacked, so the Δ*ℱr*_*k*_ is zero for these ranges.  Table 16 shows the results for malicious updates on data values of range 1. In this attack, the data values of range 1 are randomly replaced by ranges 0 and 2. This attack shows a decrease in Δ*ℱr*_*k*_ for range 1, where as the Δ*ℱr*_*k*_ for range 0 and 2 is increased. 

**Figure 6:**
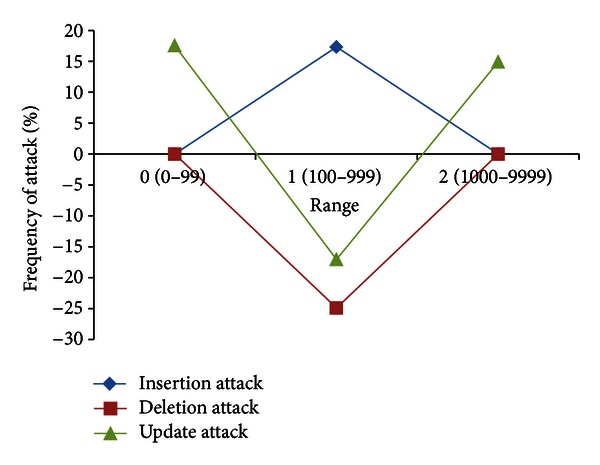
Characterization of malicious insertion, deletion, and update attacks on range 1 (100–999) of data values.

The malicious insertion, deletion, and update attacks on range 1 of data values are summarized in Figure 6. A positive increase is observed in the attacked range for insertion attack (low to high) and a negative trend (high to low) is observed in attacked range for deletion attack. The modification attack shows a negative trend (high to low) for attacked range, that is, range 1 of data values and a positive increase for nonattacked ranges, that is, range 0 and 2 of data values.

In another set of attacks, we randomly inserted, deleted, and updated 10% (lower bound) and 90% (upper bound) of tuples from the database relation *R*.  Table 17 shows the effect on fractional change in range frequency Δ*ℱr*_*k*_, for each range of data values. The fractional change in range frequency Δ*ℱr*_*k*_ has a *k* fraction of positive trend (low to high) for malicious insertion in each range of data values. Similarly, in deletion attack, a *k* fraction of negative trend (high to low) is observed for each range of data values. For example, when 10% of tuples are randomly deleted from a database relation, a decrease of approximately 10% is observed in Δ*ℱr*_*k*_ for each range of data values. The update attack does not show any specific trend as *k* fraction of different range of data values are randomly replaced by some other range of data values.

It is to be noted that the data characteristics used for our experiments like digit, length, and range of data values are cohesive to each another. Due to this relationship, we evaluated the effect of malicious data modifications on these three data characteristics. For example, if Mallory maliciously inserts a digit in a data value, the length and range of the data value are also increased. Similarly, if Mallory maliciously decreases the length of a data value, the digit count and range of the data value are also decreased (Tables 9, 13, and 17).

At the end, we summarize our findings and observations for characterization of malicious data modifications as follows.

- If there is a positive trend in fractional change Δ*ℱ* of data values in tempered database relation *R*′, it means that *ℱ* fraction of digit, range, and length of data values is maliciously inserted by Mallory in Alice's watermarked relation *R*. The characteristic of this attack is to relatively increase the low data values to high in database relation *R* (Tables 6, 10, and 14).
- If there is a negative trend in fractional change Δ*ℱ* of data values in tempered database relation *R*′, it means that *ℱ* fraction of digit, range, and length of data values is maliciously deleted by Mallory from Alice's watermarked relation *R*. The characteristic of this attack is to relatively decrease the high data values to low in database relation *R* (Tables 7, 11, and 15).
- If there is both positive and negative trends in fractional change Δ*ℱ* for digit, range, and length of data values in tempered database relation *R*′, it means that the negative trend fractional change Δ*ℱ* of data values is maliciously replaced (updated) by positive trend fractional change Δ*ℱ* of data values (Tables 8, 12, and 16).
- If there is a uniform increase of *k* in fractional change Δ*ℱ*of all data values in tempered database relation *R*′, it means that *k* fraction of similar tuples is maliciously inserted by Mallory in Alice's watermarked relation *R*. The characteristic of this attack is to relatively increase the low data values to high in database relation *R* (Tables 9, 13, and 17).
- If there is a uniform decrease of *k* in fractional change Δ*ℱ* of all data values in tempered database relation *R*′, it means that *k* fraction of tuples is maliciously deleted by Mallory from Alice's watermarked relation *R*. The characteristic of this attack is to relatively decrease the high data values to low in database relation *R* (Tables 9, 13, and 17).

## 4. Conclusions

In this paper, a fragile watermarking scheme to detect and characterize malicious tempering made in database relations is presented. The proposed scheme is based on zero watermarking approach that does not alter the database original content, and thus it overcomes the limitation of data integrity and data usability in existing watermarking schemes. In the proposed scheme, the watermarks are generated by using the local characteristics of database relation itself, like frequency distribution of various digits, lengths, and ranges of data values. This enables us to characterize the malicious modifications made to the database relations. Experimental results showed that the proposed scheme can detect and characterize malicious data modifications successfully. In the future, we intend to work on some other local characteristics of relational databases for watermark generation and to extend the proposed scheme to semifragile watermarking schemes. 

## Figures and Tables

**Algorithm 1:**
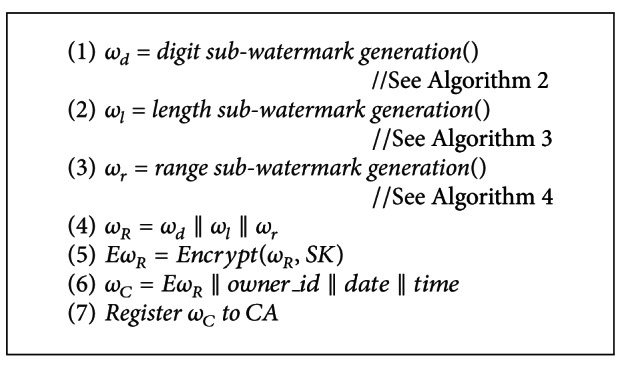
Watermark generation.

**Algorithm 2:**
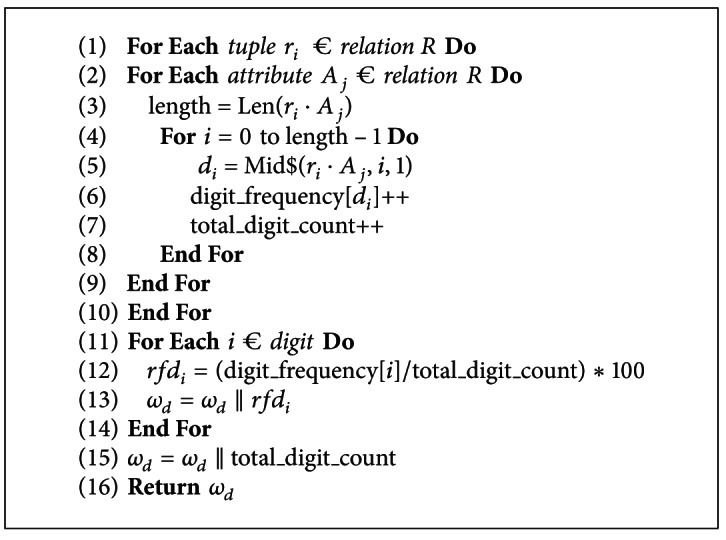
Digit sub-watermark generation.

**Algorithm 3:**
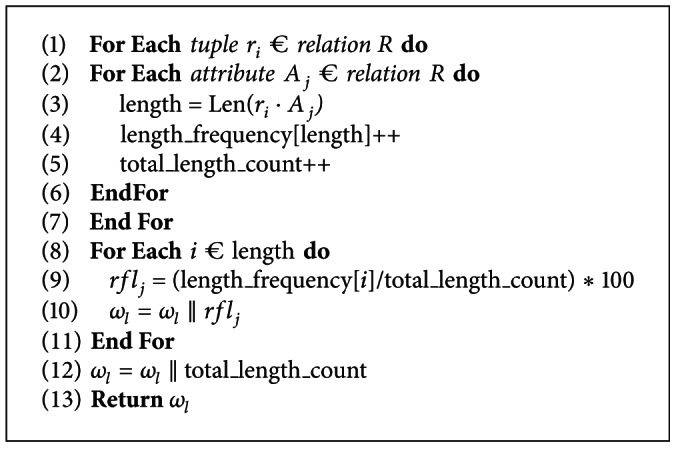
Length sub-watermark generation.

**Algorithm 4:**
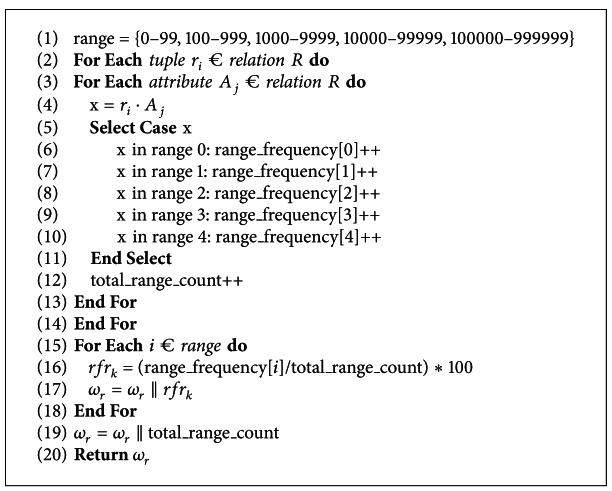
Range sub-watermark generation.

**Algorithm 5:**
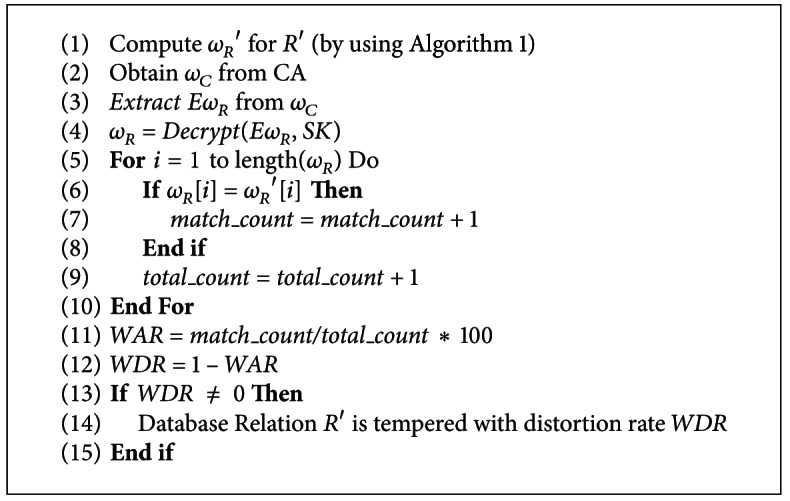
Watermark verification.

**Algorithm 6:**
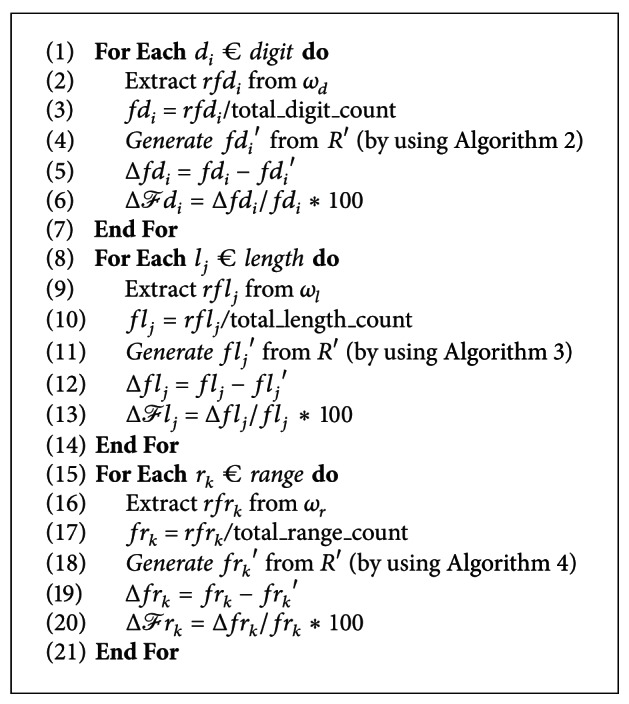
Characterization of malicious data modifications.

**Table 1:** Notations.

Symbol	Description
*R*	Database relation
PK	Primary key attribute
*r*_*i*_	The *i*th tuple
*A*_*j*_	The *j*th attribute
*η*	Number of tuples in a database relation
*ν*	Number of attributes in a database relation
*ω*_*d*_	Digit sub-watermark
*ω*_*l*_	Length sub-watermark
*ω*_*r*_	Range sub-watermark
*ω*_*R*_	Watermark for database relation *R*
*ω*_*C*_	Watermark certificate
SK	Secret key
*d*_*i*_	The *i*th digit
*l*_*j*_	The *j*th length
*r*_*k*_	The *k*th range
*fd*_*i*_	Frequency for digit *i* of data values
*fl*_*j*_	Frequency for length *j* of data values
*fr*_*k*_	Frequency for range *k* of data values
*r* *fd*_*i*_	Relative frequency for digit *i* of data values
*r* *fl*_*j*_	Relative frequency for length *j* of data values
*r* *fr*_*k*_	Relative frequency for range *k* of data values
Δ*fd*_*i*_	Change in frequency of digit *i*
Δ*fl*_*j*_	Change in frequency for length *j *
Δ*fr*_*k*_	Change in frequency for range* k *
Δ*ℱd*_*i*_	Fractional change in digit frequency for digit *i*
Δ*ℱl*_*j*_	Fractional change in length frequency for length *j *
Δ*ℱr*_*k*_	Fractional change in range frequency for range *k*
CA	Certification authority
WAR	Watermark accuracy rate
WDR	Watermark distortion rate

**Table 2:** Detection of malicious insertion of tuples with different attack rates (*η* = 10^6^).

Insertion attack rate	WAR	WDR	Temper detection
10%	18.14	81.86	Yes (High)
30%	18.56	81.44	Yes (High)
50%	20.41	79.59	Yes (High)
70%	16.67	83.33	Yes (High)
90%	16.32	83.68	Yes (High)

**Table 3:** Detection of malicious deletion of tuples with different attack rates (*η* = 10^6^).

Deletion attack rate	WAR	WDR	Temper detection
10%	24.32	75.68	Yes (High)
30%	17.88	82.12	Yes (High)
50%	20.95	79.05	Yes (High)
70%	13.08	86.92	Yes (High)
90%	14.14	85.86	Yes (High)

**Table 4:** Detection of malicious update of tuples with different attack rates (*η* = 10^6^).

Update attack rate	WAR	WDR	Temper detection
10%	20.42	79.58	Yes (High)
30%	19.89	80.11	Yes (High)
50%	19.89	80.11	Yes (High)
70%	18.94	81.06	Yes (High)
90%	14.13	85.87	Yes (High)

**Table 5:** Detection of malicious data modifications with different attack rates (*η* = 10^6^).

Insertion attack rate	Deletion attack rate	Update attack rate	WAR	WDR	Temper detection
10%	10%	10%	15.85	84.15	Yes (High)
30%	30%	30%	10.98	89.02	Yes (High)
50%	50%	50%	10.28	89.72	Yes (High)
70%	70%	70%	13.33	86.67	Yes (High)
90%	90%	90%	10.98	89.02	Yes (High)

**Table 6:** Characterization of malicious insertion attacks on digit frequency.

*d*_*i*_	*r* *fd*_*i*_	*fd*_*i*_	*r* *fd*_*i*_′	*fd*_*i*_′	Δ*fd*_*i*_	Δ*ℱd*_*i*_	Characteristic
**0**	**8.63**	**1435163**	**10.28**	**1785659**	**+350496**	**+24.42%**	↑**Low to High**
1	18.02	2995771	17.24	2995771	0	0	No change
2	19.48	3238818	18.64	3238818	0	0	No change
3	11.70	1945572	11.20	1945572	0	0	No change
4	8.22	1366062	7.86	1366062	0	0	No change
5	7.48	1244089	7.16	1244089	0	0	No change
6	6.65	1105210	6.36	1105210	0	0	No change
7	6.45	1072363	6.17	1072363	0	0	No change
8	6.60	1097669	6.32	1097669	0	0	No change
**9**	**6.76**	**1123819**	**8.78**	**1526545**	**+402726**	**+35.84%**	**↑Low to High**

**Table 7:** Characterization of malicious deletion attacks on digit frequency.

*d*_*i*_	*r* *fd*_*i*_	*fd*_*i*_	*r* *fd*_*i*_′	*fd*_*i*_′	Δ*fd*_*i*_	Δ*ℱd*_*i*_	Characteristic
**0**	**8.63**	**1435163**	**7.72**	**1256568**	**−178595**	**−12.44%**	↓**High to Low**
1	18.02	2995771	18.40	2995771	0	0	No change
2	19.48	3238818	19.89	3238818	0	0	No change
3	11.70	1945572	11.95	1945572	0	0	No change
4	8.22	1366062	8.39	1366062	0	0	No change
5	7.48	1244089	7.64	1244089	0	0	No change
6	6.65	1105210	6.79	1105210	0	0	No change
7	6.45	1072363	6.59	1072363	0	0	No change
8	6.60	1097669	6.74	1097669	0	0	No change
**9**	**6.76**	**1123819**	**5.89**	**958569**	**−165250**	**−14.70%**	↓**High to Low**

**Table 8:** Characterization of malicious update attacks on digit frequency.

*d*_*i*_	*r* *fd*_*i*_	*fd*_*i*_	*r* *fd*_*i*_′	*fd*_*i*_′	Δ*fd*_*i*_	Δ*ℱd*_*i*_	Characteristic
**0**	**8.63**	**1435163**	**5.71**	**948993**	**−486170**	**−33.88%**	**↓High to Low**
1	18.02	2995771	19.01	3159784	+164013	+5.47%	↑Low to High
2	19.48	3238818	20.97	3485451	+246633	+7.61%	↑Low to High
3	11.70	1945572	11.91	1980325	+34753	+1.79%	↑Low to High
4	8.22	1366062	8.52	1416889	+50827	+3.72%	↑Low to High
5	7.48	1244089	8.22	1365803	+121714	+9.78%	↑Low to High
6	6.65	1105210	6.90	1146565	+41355	+3.74%	↑Low to High
7	6.45	1072363	7.14	1187586	+115223	+10.74%	↑Low to High
8	6.60	1097669	6.78	1127651	+29982	+2.73%	↑Low to High
**9**	**6.76**	**1123819**	**4.85**	**805489**	**−318330**	**−28.33%**	**↓High to Low**

**Table 9:** Characterization of malicious modifications on digit frequency.

	Insertion attack		Deletion attack		Update attack	
Attack rate	10%	90%	10%	90%	10%	90%
*d*_*i*_	Δ*ℱd*_*i*_%	Δ*ℱd*_*i*_%	Δ*ℱd*_*i*_%	Δ*ℱd*_*i*_%	Δ*ℱd*_*i*_%	Δ*ℱd*_*i*_%
0	+9.51	+90.77%	−9.63%	−89.65%	+0.41	+0.45
1	+10.51	+94.47%	−10.11%	−89.05%	+0.80	+15.91
2	+10.49	+90.14%	−10.67%	−90.20%	−0.57	−3.72
3	+9.63	+94.72%	−9.87%	−90.30%	−2.12	+3.39
4	+9.47	+83.78%	−10.07%	−90.82%	−0.28	−7.34
5	+9.01	+82.00%	−9.43%	−91.26%	+0.40	−11.43
6	+9.08	+81.84%	−9.48%	−91.01%	+0.13	−7.78
7	+10.17	+88.20%	−9.95%	−89.83%	+0.05	+1.46
8	+10.23	+88.86%	−10.06%	−89.89%	−0.22	+2.16
9	+10.25	+88.36%	−10.37%	−90.66%	−0.96	−2.62

The detailed experiments for this set of attacks are presented in the Appendix (Tables 18(a)–18(f)).

**Table 10:** Characterization of malicious insertion attacks on length of data values.

*l*_*j*_	*r* *fl*_*j*_	*fl*_*j*_	*r* *fl*_*j*_′	*fl*_*j*_′	Δ*fl*_*j*_	Δ*ℱl*_*j*_	Characteristic
1	5.74	325894	5.27	325894	0	0	No change
2	20.19	1146469	18.54	1146469	0	0	No change
**3**	**48.66**	**2762791**	**52.85**	**3267609**	**+504818**	**+18.27%**	**↑Low to High**
4	25.41	1442906	23.34	1442906	0	0	No change

**Table 11:** Characterization of malicious deletion attacks on length of data values.

*l*_*j*_	*r* *fl*_*j*_	*fl*_*j*_	*r* *fl*_*j*_′	*fl*_*j*_′	Δ*fl*_*j*_	Δ*ℱl*_*j*_	Characteristic
1	5.74	325894	6.52	325894	0	0.00	No change
2	20.19	1146469	22.92	1146469	0	0.00	No change
**3**	**48.66**	**2762791**	**41.71**	**2085761**	**−677030**	**−24.51%**	**↓High to Low**
4	25.41	1442906	28.85	1442906	0	0.00	No change

**Table 12:** Characterization of malicious update attacks on length of data values.

*l*_*j*_	*r* *fl*_*j*_	*fl*_*j*_	*r* *fl*_*j*_′	*fl*_*j*_′	Δ*fl*_*j*_	Δ*ℱl*_*j*_	Characteristic
1	5.74	325894	6.31	358142	+32248	+9.90%	↑Low to High
2	20.19	1146469	27.57	1565462	+418993	+36.55%	↑Low to High
**3**	**48.66**	**2762791**	**34.27**	**1945657**	**−817134**	**−29.58%**	**↓High to Low**
4	25.41	1442906	31.86	1808799	+365893	+25.36%	↑Low to High

**Table 13:** Characterization of malicious modifications on length of data values.

	Insertion attack		Deletion attack		Update attack	
Attack rate	10%	90%	10%	90%	10%	90%
*l*_*j*_	Δ*ℱl*_*j*_%	Δ*ℱl*_*j*_%	Δ*ℱl*_*j*_%	Δ*ℱl*_*j*_%	Δ*ℱl*_*j*_%	Δ*ℱl*_*j*_%
1	+9.55	100.58	−8.94	−91.25	−3.65	−38.67
2	+10.20	89.64	−10.21	−89.28	1.89	6.13
3	+10.35	89.91	−9.98	−88.92	1.57	11.03
4	+9.41	87.71	−10.16	−91.93	−2.94	−12.33

The detailed experiments for this set of attacks are presented in the Appendix (Tables 19(a)–19(f)).

**Table 14:** Characterization of malicious insertion attacks on range of data values.

Range	*r*_*k*_	*r* *fr*_*k*_	*fr*_*k*_	*r* *fr*_*k*_′	*fr*_*k*_′	Δ*fr*_*k*_	Δ*ℱr*_*k*_	Characteristic
0	0–99	25.59	1436986	23.60	1436986	0	0	No change
**1**	**100–999**	**48.73**	**2736731**	**52.72**	**3210988**	**+474257**	**+17.33%**	↑**Low to High**
2	1000–9999	25.68	1442223	23.68	1442223	0	0	No change

**Table 15:** Characterization of malicious deletion attacks on range of data values.

Range	*r*_*k*_	*r* *fr*_*k*_	*fr*_*k*_	*r* *fr*_*k*_′	*fr*_*k*_′	Δ*fr*_*k*_	Δ*ℱr*_*k*_	Characteristic
0	0–99	25.59	1436986	29.12	1436986	0	0	No change
**1**	**100–999**	**48.73**	**2736731**	**41.65**	**2054875**	**−681856**	**−24.92%**	↓**High to Low**
2	1000–9999	25.68	1442223	29.23	1442223	0	0	No change

**Table 16:** Characterization of malicious update attacks on range of data values.

Range	*r*_*k*_	*r* *fr*_*k*_	*fr*_*k*_	*r* *fr*_*k*_′	*fr*_*k*_′	Δ*fr*_*k*_	Δ*ℱr*_*k*_	Characteristic
0	0–99	25.59	1436986	30.07	1688965	251979	+17.54%	↑Low to High
**1**	**100–999**	**48.73**	**2736731**	**40.42**	**2269854**	**−466877**	**−17.06%**	**↓High to Low**
2	1000–9999	25.68	1442223	29.51	1657121	214898	+14.91%	↑Low to High

**Table 17:** Characterization of malicious data modifications on range of data values.

		Insertion attack		Deletion attack		Update attack	
Attack rate			10%	90%	10%	90%	10%
Range	*r*_*k*_	Δ*ℱr*_*k*_%	Δ*ℱr*_*k*_%	Δ*ℱr*_*k*_%	Δ*ℱr*_*k*_ *% *	Δ*ℱr*_*k*_%	Δ*ℱr*_*k*_%
0	0–99	+9.97	+92.33	−9.84	−89.76	0.39	−5.91
1	100–999	+10.34	+89.86	−9.99	−88.90	1.48	10.46
2	1000–9999	+9.41	+87.71	−10.16	−91.93	−2.94	−12.41

*The detailed experiments for this set of attacks are presented in the Appendix (Tables 20(a)–20(f)).

**Table:** (a) Characterization of malicious insertion attacks on digit frequency (*η* = 10^6^, attack rate = 10%)

*d*_*i*_	*rf* *d*_*i*_	*fd*_*i*_	*rf* *d*_*i*_′	*fd*_*i*_′	Δ*fd*_*i*_	Δ*ℱd*_*i*_	Characteristic
0	8.44	247345	8.40	270858	23513	+9.51%	↑Low to High
1	16.28	477362	16.37	527535	50173	+10.51%	↑Low to High
2	19.88	582616	19.97	643745	61129	+10.49%	↑Low to High
3	11.37	333378	11.34	365471	32093	+9.63%	↑Low to High
4	8.83	258748	8.79	283251	24503	+9.47%	↑Low to High
5	8.31	243554	8.24	265510	21956	+9.01%	↑Low to High
6	7.14	209399	7.09	228418	19019	+9.08%	↑Low to High
7	6.40	187586	6.41	206659	19073	+10.17%	↑Low to High
8	6.49	190268	6.51	209737	19469	+10.23%	↑Low to High
9	6.86	201050	6.88	221653	20603	+10.25%	↑Low to High

**Table:** (b) Characterization of malicious insertion attacks on digit frequency (*η* = 10^6^, attack rate = 90%)

*d*_*i*_	*rf* *d*_*i*_	*fd*_*i*_	*rf* *d*_*i*_′	*fd*_*i*_′	Δ*fd*_*i*_	Δ*ℱd*_*i*_	Characteristic
0	8.44	247345	8.51	471852	224507	+90.77%	↑Low to High
1	16.28	477362	16.73	928331	450969	+94.47%	↑Low to High
2	19.88	582616	19.97	1107797	525181	+90.14%	↑Low to High
3	11.37	333378	11.70	649150	315772	+94.72%	↑Low to High
4	8.83	258748	8.57	475532	216784	+83.78%	↑Low to High
5	8.31	243554	7.99	443279	199725	+82.00%	↑Low to High
6	7.14	209399	6.86	380768	171369	+81.84%	↑Low to High
7	6.40	187586	6.36	353031	165445	+88.20%	↑Low to High
8	6.49	190268	6.48	359339	169071	+88.86%	↑Low to High
9	6.86	201050	6.83	378696	177646	+88.36%	↑Low to High

**Table:** (c) Characterization of malicious deletion attacks on digit frequency (*η* = 10^6^, attack rate = 10%)

*d*_*i*_	*rf* *d*_*i*_	*fd*_*i*_	*rf* *d*_*i*_′	*fd*_*i*_′	Δ*fd*_*i*_	Δ*ℱd*_*i*_	Characteristic
0	8.44	247345	8.48	223528	−23817	−9.63%	↓High to Low
1	16.28	477362	16.27	429114	−48248	−10.11%	↓High to Low
2	19.88	582616	19.74	520475	−62141	−10.67%	↓High to Low
3	11.37	333378	11.40	300483	−32895	−9.87%	↓High to Low
4	8.83	258748	8.83	232703	−26045	−10.07%	↓High to Low
5	8.31	243554	8.37	220577	−22977	−9.43%	↓High to Low
6	7.14	209399	7.19	189555	−19844	−9.48%	↓High to Low
7	6.40	187586	6.41	168921	−18665	−9.95%	↓High to Low
8	6.49	190268	6.49	171131	−19137	−10.06%	↓High to Low
9	6.86	201050	6.83	180196	−20854	−10.37%	↓High to Low

**Table:** (d) Characterization of malicious deletion attacks on digit frequency (*η* = 10^6^, attack rate = 90%)

*d*_*i*_	*rf* *d*_*i*_	*fd*_*i*_	*rf* *d*_*i*_′	*fd*_*i*_′	Δ*fd*_*i*_	Δ*ℱd*_*i*_	Characteristic
0	8.44	247345	8.89	25612	−221733	−89.65%	↓High to Low
1	16.28	477362	18.14	52276	−425086	−89.05%	↓High to Low
2	19.88	582616	19.80	57072	−525544	−90.20%	↓High to Low
3	11.37	333378	11.22	32342	−301036	−90.30%	↓High to Low
4	8.83	258748	8.24	23758	−234990	−90.82%	↓High to Low
5	8.31	243554	7.38	21281	−222273	−91.26%	↓High to Low
6	7.14	209399	6.53	18815	−190584	−91.01%	↓High to Low
7	6.40	187586	6.62	19071	−168515	−89.83%	↓High to Low
8	6.49	190268	6.67	19239	−171029	−89.89%	↓High to Low
9	6.86	201050	6.52	18780	−182270	−90.66%	↓High to Low

**Table:** (e) Characterization of malicious update attacks on digit frequency (*η* = 10^6^, attack rate = 10%)

*d*_*i*_	*rf* *d*_*i*_	*fd*_*i*_	*rf* *d*_*i*_′	*fd*_*i*_′	Δ*fd*_*i*_	Δ*ℱd*_*i*_	Characteristic
0	8.438048	247345	8.49	248357	1012	+0.41%	↑Low to High
1	16.28496	477362	16.46	481186	3824	+0.80%	↑Low to High
2	19.87565	582616	19.81	579315	−3301	−0.57%	↓High to Low
3	11.37302	333378	11.16	326311	−7067	−2.12%	↓High to Low
4	8.827055	258748	8.82	258020	−728	−0.28%	↓High to Low
5	8.30872	243554	8.36	244519	965	+0.40%	↑Low to High
6	7.143539	209399	7.17	209680	281	+0.13%	↑Low to High
7	6.3994	187586	6.42	187678	92	+0.05%	↑Low to High
8	6.490895	190268	6.49	189845	−423	−0.22%	↓High to Low
9	6.858718	201050	6.81	199111	−1939	−0.96%	↓High to Low

**Table:** (f) Characterization of malicious update attacks on digit frequency (*η* = 10^6^, attack rate = 90%)

*d*_*i*_	*rf* *d*_*i*_	*fd*_*i*_	*rf* *d*_*i*_′	*fd*_*i*_′	Δ*fd*_*i*_	Δ*ℱd*_*i*_	Characteristic
0	8.438048	247345	8.46	248462	1117	+0.45%	↑Low to High
1	16.28496	477362	18.84	553334	75972	+15.91%	↑Low to High
2	19.87565	582616	19.10	560965	−21651	−3.72%	↓High to Low
3	11.37302	333378	11.74	344665	11287	+3.39%	↑Low to High
4	8.827055	258748	8.16	239767	−18981	−7.34%	↓High to Low
5	8.30872	243554	7.35	215727	−27827	−11.43%	↓High to Low
6	7.143539	209399	6.58	193116	−16283	−7.78%	↓High to Low
7	6.3994	187586	6.48	190330	2744	+1.46%	↑Low to High
8	6.490895	190268	6.62	194384	4116	+2.16%	↑Low to High
9	6.858718	201050	6.67	195785	−5265	−2.62%	↓High to Low

**Table:** (a) Characterization of malicious insertion attacks on length of data values (*η* = 10^6^, attack rate = 10%)

*l*_*j*_	*rf* *l*_*j*_	*fl*_*j*_	*rf* *l*_*j*_′	*fl*_*j*_′	Δ*fl*_*j*_	Δ*ℱl*_*j*_	Characteristic
1	7.11	71085	7.08	77875	6790	+9.55%	↑Low to High
2	19.76	197605	19.80	217767	20162	+10.20%	↑Low to High
3	45.18	451787	45.32	498531	46744	+10.35%	↑Low to High
4	27.95	279523	27.80	305827	26304	+9.41%	↑Low to High

**Table:** (b) Characterization of malicious insertion attacks on length of data values (*η* = 10^6^, attack rate = 90%)

*l*_*j*_	*rf* *l*_*j*_	*fl*_*j*_	*rf* *l*_*j*_′	*fl*_*j*_′	Δ*fl*_*j*_	Δ*ℱl*_*j*_	Characteristic
1	7.11	71085	7.50	142584	71499	+100.58%	↑Low to High
2	19.76	197605	19.72	374737	177132	+89.64%	↑Low to High
3	45.18	451787	45.16	857986	406199	+89.91%	↑Low to High
4	27.95	279523	27.62	524693	245170	+87.71%	↑Low to High

**Table:** (c) Characterization of malicious deletion attacks on length of data values (*η* = 10^6^, attack rate = 10%)

*l*_*j*_	*rf* *l*_*j*_	*fl*_*j*_	*rf* *l*_*j*_′	*fl*_*j*_′	Δ*fl*_*j*_	Δ*ℱl*_*j*_	Characteristic
1	7.11	71085	7.19	64733	−6352	−8.94%	↓High to Low
2	19.76	197605	19.71	177421	−20184	−10.21%	↓High to Low
3	45.18	451787	45.19	406721	−45066	−9.98%	↓High to Low
4	27.95	279523	27.90	251125	−28398	−10.16%	↓High to Low

**Table:** (d) Characterization of malicious deletion attacks on length of data values (*η* = 10^6^, attack rate = 90%)

*l*_*j*_	*rf* *l*_*j*_	*fl*_*j*_	*rf* *l*_*j*_′	*fl*_*j*_′	Δ*fl*_*j*_	Δ*ℱl*_*j*_	Characteristic
1	7.11	71085	6.22	6221	−64864	−91.25%	↓High to Low
2	19.76	197605	21.19	21186	−176419	−89.28%	↓High to Low
3	45.18	451787	50.04	50039	−401748	−88.92%	↓High to Low
4	27.95	279523	22.55	22554	−256969	−91.93%	↓High to Low

**Table:** (e) Characterization of malicious update attacks on length of data values (*η* = 10^6^, attack rate = 10%)

*l*_*j*_	*rf* *l*_*j*_	*fl*_*j*_	*rf* *l*_*j*_′	*fl*_*j*_′	Δ*fl*_*j*_	Δ*ℱl*_*j*_	Characteristic
1	7.11	71085	6.85	68488	−2597	−3.65%	↓High to Low
2	19.76	197605	20.13	201333	3728	+1.89%	↑Low to High
3	45.18	451787	45.89	458869	7082	+1.57%	↑Low to High
4	27.95	279523	27.13	271310	−8213	−2.94%	↓High to Low

**Table:** (f) Characterization of malicious update attacks on length of data values (*η* = 10^6^, attack rate = 90%)

*l*_*j*_	*rf* *l*_*j*_	*fl*_*j*_	*rf* *l*_*j*_′	*fl*_*j*_′	Δ*fl*_*j*_	Δ*ℱl*_*j*_	Characteristic
1	7.11	71085	4.36	43599	−27486	−38.67%	↓High to Low
2	19.76	197605	20.97	209711	12106	+6.13%	↑Low to High
3	45.18	451787	50.16	501622	49835	+11.03%	↑Low to High
4	27.95	279523	24.51	245068	−34455	−12.33%	↓High to Low

**Table:** (a) Characterization of malicious insertion attacks on range of data values (*η* = 10^6^, attack rate = 10%)

Range No	*r*_*k*_	*rf* *r*_*k*_	*fr*_*k*_	*rf* *r*_*k*_′	*fr*_*k*_′	Δ*fr*_*k*_	Δ*ℱr*_*k*_	Characteristic
0	0–99	25.59	1436986	26.49	288506	26164	+9.97%	↑Low to High
1	100–999	48.73	2736731	45.43	494709	46373	+10.34%	↑Low to High
2	1000–9999	25.68	1442223	28.08	305806	26304	+9.41%	↑Low to High

**Table:** (b) Characterization of malicious insertion attacks on range of data values (*η* = 10^6^, attack rate = 90%)

Range No	*r*_*k*_	*rf* *r*_*k*_	*fr*_*k*_	*rf* *r*_*k*_′	*fr*_*k*_′	Δ*fr*_*k*_	Δ*ℱr*_*k*_	Characteristic
0	0–99	25.59	1436986	26.83	504566	242224	+92.33%	↑Low to High
1	100–999	48.73	2736731	45.27	851193	402857	+89.86%	↑Low to High
2	1000–9999	25.68	1442223	27.90	524663	245161	+87.71%	↑Low to High

**Table:** (c) Characterization of malicious deletion attacks on range of data values (*η* = 10^6^, attack rate = 10%)

Range No	*r*_*k*_	*rf* *r*_*k*_	*fr*_*k*_	*rf* *r*_*k*_′	*fr*_*k*_′	Δ*fr*_*k*_	Δ*ℱr*_*k*_	Characteristic
0	0–99	25.59	1436986	26.54	236532	−25810	−9.84%	↓High to Low
1	100–999	48.73	2736731	45.28	403562	−44774	−9.99%	↑Low to High
2	1000–9999	25.68	1442223	28.18	251104	−28398	−10.16%	↓High to Low

**Table:** (d) Characterization of malicious deletion attacks on range of data values (*η* = 10^6^, attack rate = 90%)

Range No	*r*_*k*_	*rf* *r*_*k*_	*fr*_*k*_	*rf* *r*_*k*_′	*fr*_*k*_′	Δ*fr*_*k*_	Δ*ℱr*_*k*_	Characteristic
0	0–99	25.59	1436986	27.08	26857	−235485	−89.76%	↓High to Low
1	100–999	48.73	2736731	50.18	49757	−398579	−88.90%	↑Low to High
2	1000–9999	25.68	1442223	22.74	22547	−256955	−91.93%	↓High to Low

**Table:** (e) Characterization of malicious update attacks on range of data values (*η* = 10^6^, attack rate = 10%)

Range No	*r*_*k*_	*rf* *r*_*k*_	*fr*_*k*_	*rf* *r*_*k*_′	*fr*_*k*_′	Δ*fr*_*k*_	Δ*ℱr*_*k*_	Characteristic
0	0–99	25.59	1436986	26.61	263370	1028	+0.39%	↑Low to High
1	100–999	48.73	2736731	45.97	454956	6620	+1.48%	↑Low to High
2	1000–9999	25.68	1442223	27.41	271271	−8231	−2.94%	↓High to Low

**Table:** (f) Characterization of malicious update attacks on range of data values (*η* = 10^6^, attack rate = 90%)

Range No	*r*_*k*_	*rf* *r*_*k*_	*fr*_*k*_	*rf* *r*_*k*_′	*fr*_*k*_′	Δ*fr*_*k*_	Δ*ℱr*_*k*_	Characteristic
0	0–99	25.59	1436986	25.01	246838	−15504	−5.91%	↓High to Low
1	100–999	48.73	2736731	50.18	495248	46912	+10.46%	↑Low to High
2	1000–9999	25.68	1442223	24.81	244819	−34683	−12.41%	↓High to Low

## Appendix

- Characterization of malicious attacks on digit frequency (deterministic) (see Table 18).
- Characterization of malicious attacks on length of data values (deterministic) (see Table 19).
- Characterization of malicious attacks on range of data values (deterministic) (see Table 20).
